# Aerobic exercise-induced circulating extracellular vesicle combined decellularized dermal matrix hydrogel facilitates diabetic wound healing by promoting angiogenesis

**DOI:** 10.3389/fbioe.2022.903779

**Published:** 2022-08-23

**Authors:** Haifeng Liu, Bing Wu, Xin Shi, Yanpeng Cao, Xin Zhao, Daqiang Liang, Qihuang Qin, Xinzhi Liang, Wei Lu, Daping Wang, Jun Liu

**Affiliations:** ^1^ Guangzhou Medical University, Guangzhou, China; ^2^ Department of Sports Medicine, The First Affiliated Hospital of Shenzhen University, Shenzhen Second People’s Hospital, Shenzhen, China; ^3^ Department of Limbs (Foot and Hand) Microsurgery, Affiliated Chenzhou Hospital, Hengyang Medical School, University of South China, Chenzhou, China

**Keywords:** extracellular vesicles, aerobic exercise, diabetes, angiogenesis, wound healing

## Abstract

**Background:** Insufficient blood supply results in unsatisfactory wound healing, especially for challenging wound repair such as diabetic wound defects. Regular exercise training brings a lot of benefits to cardiovascular fitness and metabolic health including attenuation of T2DM progression. Circulating extracellular vesicles (EVs) are postulated to carry a variety of signals involved in tissue crosstalk by their modified cargoes, representing novel mechanisms for the effects of exercise. Prominently, both acute and chronic aerobic exercise training can promote the release of exercise-induced cytokines and enhance the angiogenic function of circulating angiogenic cell–derived EVs.

**Methods:** We investigated the possible angiogenesis potential of aerobic exercise-induced circulating EVs (EXE-EVs) on diabetic wound healing. Circulating EVs were isolated from the plasma of rats subjected to 4 weeks of moderate aerobic exercise or sedentariness 24 h after the last training session. The therapeutic effect of circulating EVs was evaluated *in vitro* by proliferation, migration, and tube formation assays of human umbilical vein endothelial cells (HUVECs), as well as *in vivo* by quantification of angiogenesis and cutaneous wound healing in diabetic rats.

**Results:** The number of circulating EVs did not change significantly in exercised rats 24 h post-exercise in comparison with the sedentary rats. Nevertheless, EXE-EVs showed remarkable pro-angiogenic effect by augmenting proliferation, migration, and tube formation of HUVECs. Furthermore, the findings of animal experiments revealed that the EXE-EVs delivered by decellularized dermal matrix hydrogel (DDMH) could significantly promote the repair of skin defects through stimulating the regeneration of vascularized skin.

**Discussion:** The present study is the first attempt to demonstrate that aerobic exercise-induced circulating EVs could be utilized as a cell-free therapy to activate angiogenesis and promote diabetic wound healing. Our findings suggest that EXE-EVs may stand for a potential strategy for diabetic soft tissue wound repair.

## 1 Introduction

Diabetic skin wounds or ulcers have been recognized as the frequently seen co-morbidities related to diabetes mellitus. Diabetic skin ulcers are usually manifested as unhealing sores with disintegrated dermal tissues including epidermis, dermis, or subcutaneous tissue, which make them hard to be fully repaired. Although a lot of efforts have been made for diabetic skin wounds, the recovery rates remain poor, with nearly 28% of cases receiving lower extremity amputation (Bakker et al., 2016). What is worse, the amputation-related death rate is as high as 50%–59% 5 years after amputation (Armstrong and Lavery, 1998; Alavi et al., 2014; Armstrong et al., 2017; Grennan, 2019). Poor vascular supply is an important factor related to diabetic ulceration progress. It has been demonstrated that the diabetic state induces diverse angiogenic defects during early and late wound healing periods, which impact vessel growth and maturation (Prompers et al., 2008; Okonkwo and DiPietro, 2017).

Exercise contributes to healthier global status for both ill and healthy people. The benefits contain increasing lifespan, delaying the occurrence of aging-related diseases, ameliorating the onset and intensity of contracting communicable diseases, and reducing the risk of a multitude of disorders from metabolic diseases to cancer (Baik et al., 2000; Ekelund et al., 2015; Hills, 2018; Northey et al., 2018; de Meireles et al., 2019; Pedersen, 2019; Radak et al., 2019; Semeraro et al., 2020). The involvement of resistance or aerobic exercise has been extensively accepted as an effective prevention and treatment approach for diabetes mellitus (Warburton and Bredin, 2017). Although it is complicated and involves multi-factors to provide such distinct health benefits, exercise has been demonstrated to trigger the release of a variety of cytokines, peptides, and proteins, termed “exerkines,” into the circulation which can be contained within extracellular vesicles (EVs) (Görgens et al., 2015; Lombardi et al., 2016).

EVs are a heterogeneous group of endogenous membrane vesicles secreted by almost all cell types which serve an important function as mediators of intercellular communication and crosstalk between organs through the transmission of various signaling molecules (Todorova et al., 2017; Shah et al., 2018). Exercise-derived EVs are hypothesized to be released from cells to the blood and can confer the systemic benefits of exercise to distal organs (Safdar et al., 2016). As key players in aerobic exercise-mediated beneficial effects, circulating EVs have been shown to protect the heart against ischemia injury (Hou et al., 2019). Moreover, some studies have demonstrated that both acute and chronic aerobic exercise training show apparent benefits on pro-angiogenic potential of circulating angiogenic cells (CACs) (Evans et al., 2020). These findings indicate that the circulating EVs may exert a vital function in exercise-conferred vascularization benefits.

To investigate the effect of aerobic exercise-induced circulating EVs (EXE-EVs) on the construction of vascularized skin tissue, EXE-EVs and human umbilical vein endothelial cells (HUVECs) were co-cultured to determine whether it can facilitate angiogenesis *in vitro*. Furthermore, the EXE-EVs combined with dermal extracellular matrix hydrogel (DDMH) material were applied to repair diabetic cutaneous wounds in rats and analyzed by gross, histology, radiography, and immunofluorescence assays. This study may offer a promising cell-free biotherapy strategy for diabetic cutaneous wound healing.

## 2 Materials and methods

### 2.1 Animals

Our animal protocols of Sprague-Dawley (SD) rats gained approval from the Animal Ethics Committee of Chenzhou No. 1 People’s Hospital. The 6-week-old SD rats weighing 250–300 g were kept in the standard environment of 22 ± 2°C and the 12-h/12-h light/dark cycle and were allowed to drink water and eat food freely. All animal care protocols and surgical procedures were carried out following the Guide for the Care and Use of Laboratory Animals released by US National Institutes of Health (Publication No. 85-23, revised 1996). The rat numbers in different experiments are presented in the figures.

### 2.2 Exercise protocol

The rats were randomly assigned to the aerobic exercise (EXE) group and the sedentary (SED) group. Aerobic exercise training consisted of a group of treadmill running sessions which were performed on a motorized rodent treadmill (ZH-PT; Anhui Zhenghua Co., Ltd., China). The running protocol was modified from a previously published procedure (Arida et al., 1999; Elsner et al., 2011). To be specific, 20 min of daily running for a 4-week period as a moderate treadmill running protocol (20 min of running each day for 4 weeks) was used, in which all rats ran at 60% of their maximal oxygen uptake VO_2_ (60% VO_2 peak)_, which was determined indirectly prior to training (Brooks and White, 1978; Elsner et al., 2013; Lovatel et al., 2013). In the first week, treadmill speed was increased at a low initial speed daily until all mice tolerated running at 60% of VO_2 peak_ for a 20-min period daily. For rats refusing to run, their backs were gently tapped to encourage running. Rats in the SED group were left on the motionless treadmill for 20 min daily without any stimulus to run for 4 weeks. All procedures were conducted during 15:00–18:00.

### 2.3 Isolation, identification, and labeling of circulating extracellular vesicles

#### 2.3.1 Isolation of circulating extracellular vesicles

Blood was collected in EXE and SED rats 24 h after the final training session. After being centrifuged at 1,600 × g for 20 min at 4°C, the obtained plasma was subjected to 30 min of centrifugation at 10,000 × g at 4°C to remove cells and platelets. 0.22-mm filters (BD Biosciences, San Jose, United States) were utilized to filter supernatants for eliminating cell debris. Thereafter, the supernatants were subjected to ultracentrifugation at 100,000 × g for 4 h at 4°C. After washing with phosphate-buffered saline (PBS) at 100,000 × g for 20 min, the EV-containing pellet was resuspended in the right amount of PBS. The acquired plasma EVs from sedentary rats (SED-EVs) or exercise rats (EXE-EVs) were stored at –80°C for later experiments, which were used as soon as possible (within 7 days) and avoided freeze–thaw cycles.

#### 2.3.2 Identification of circulating extracellular vesicles

For identification of circulating EVs, EV morphology was analyzed using a transmission electron microscope (TEM, Hitachi, Tokyo, Japan), and a ZetaView PMX 120 (Particle Metrix, Inning am Ammersee, Germany) was used for nanoparticle tracking analysis (NTA) to analyze particle size distribution. Additionally, the specific EV surface markers (CD9, CD63, Calnexin, and TSG101) were measured through Western blotting (WB) assay. The BCA quantitation kit (Beyotime, Shanghai, China) was adopted for determining protein content in EV suspension. Primary antibodies used in this study were as follows: anti-CD63 (1:500; Abcam, Cambridge, Britain), anti-CD9 (1:500; Abcam), anti-TSG101 (1:500; Abcam), and anti-Calnexin (1:500; Abcam). For the *in vitro* experiments, three independent repeated tests were performed (*n* = 3).

#### 2.3.3 Labeling of circulating extracellular vesicles

To determine the internalization of circulating EVs by HUVECs, a PKH26 red fluorescent cell linker kit (Sigma-Aldrich, St. Louis, United States) was utilized to pre-stain EVs according to the manufacturer’s instructions. Meanwhile, DiR stains (40757ES25, Yeasen, Shanghai, China) were used for staining EVs to track the location of circulating EVs *in vivo*. The labeled EVs were rinsed in PBS and collected by ultracentrifugation (100,000 × g for 30 min) at 4°C (Dong et al., 2018). Then, the labeled EVs were resuspended in PBS and used for the next experiments.

### 2.4 Vascular endothelial growth factor expression in circulating extracellular vesicles

The expression of vascular endothelial growth factor (VEGF), which has been proved to induce angiogenesis and the formation of granulation tissue (Testori et al., 2011), was detected in both EXE-EVs and SED-EVs using WB assay. Briefly, total proteins of EVs were extracted using RIPA lysis buffer supplemented with a protease inhibitor (Sigma). Then, sodium dodecyl sulfate-polyacrylamide gel electrophoresis (SDS-PAGE) was used to separate EV protein, followed by protein transfer onto polyvinylidene fluoride membranes (Millipore, Darmstadt, Germany). After blocking with 5% non-fat milk, the membranes were incubated with primary VEGF antibodies (1:500, Proteintech, Wuhan, China) overnight at 4°C. Then, the membranes were washed and incubated with horseradish peroxidase-conjugated secondary antibodies for 1 h at room temperature. After reaction with a chemiluminescence reagent (Thermo Fisher Scientific, Waltham, United States) for 1 min, the bands were imaged using a ChemiDoc XRS Plus luminescent image analyzer (Bio-Rad, Hercules, United States). The relative protein expression was analyzed using ImageJ software (Version 1.8.0, National Institutes of Health, United States).

## 3 Impact of circulating extracellular vesicles on endothelial cell angiogenesis *in vitro*

### 3.1 Human umbilical vein endothelial cells culture

HUVEC line c-12,206 was procured from the Cell Bank of the Chinese Academy of Sciences (Shanghai, China). HUVECs were cultivated within a high-glucose (HG) DMEM complete medium (Gibco, Grand Island, United States) that contained 1% penicillin-streptomycin (Gibco) as well as 10% fetal bovine serum (FBS; Gibco). HUVECs were incubated in 5% CO2 at 37°C. FBS was substituted by EV-free serum in the subsequent assays.

### 3.2 Extracellular vesicle uptake by human umbilical vein endothelial cells

HUVECs were incubated with the PKH26-labeled circulating EVs (50 μg/ml) for 12 h at 37°C in the dark (Ti et al., 2015; Wang et al., 2020). After washing with PBS, cells were fixed in 4% paraformaldehyde for 10 min and rinsed in PBS thrice. Then, cell nuclei were stained with DAPI (0.5 μg/ml; Invitrogen, Carlsbad, United States), and cellular uptake of EVs was observed using a Zeiss AxioImager.M2 fluorescence microscope (Zeiss, Solms, Germany) and the fluorescence signal was analyzed using the Apotome.2 System (Zeiss).

### 3.3 Cell proliferation assay

The effect of circulating EVs on proliferation of HUVECs was determined by Cell Counting Kit-8 assay (CCK-8, 70-CCK8100, MultiSciences, Hangzhou, China) according to the manufacturer’s instructions. Briefly, HUVECs were inoculated in 96-well plates at 4 × 10^3^/well, followed by culture within complete medium that contained 10 μg/well EXE-EVs or SED-EVs (Hu et al., 2018), with three repeated wells per group. A group without cells served as the blank. Fresh culture medium was replaced every day. The absorbance tests were performed on days 1, 2, 3, 4, and 5. Before testing, 10 μL CCK-8 solution was added to all wells and incubated at 37°C for 1 h. Then, the optical density (OD) was measured using a microplate reader (Bio-Rad 680, Bio-Rad, Hercules, CA, United States) at 450 nm of each well. OD values of tested wells were subtracted from those of blank wells to determine cell survival/proliferation.

### 3.4 Cell migration assay

HUVEC migration was assessed using scratch and transwell assay. In scratch assay, the fused monolayer of HUVECs was scratched on a 6-well plate (three replicates per group) using a 200-μL pipette tip. Then, detached cells and cell debris were removed by rinsing with PBS. Afterwards, complete medium that contained SED-EVs or EXE-EVs (100 μg/well) was supplemented to culture cells (Wang et al., 2020). HUVECs cultured in complete medium without EV stimulation were used as the negative control. The scratch wound was visualized using an optical microscope (Leica DMI6000B, Leica, Germany) at 0/6/12 h after wound formation. Then, ImageJ software was utilized to measure the wound scratch area. This work determined the wound closure area by migration area (%) = (A_0_ –A_n_)/A_0_ × 100, with A_0_ representing the original wound area and A_n_ representing the rest wound area when photographing (Hu et al., 2018).

Transwell assay was performed using 24-well Transwell inserts (Costar 3422, Corning, United States) with three repeated wells per group. Briefly, complete medium with SED-EVs or EXE-EVs (50 μg/well) was added to the lower chamber of the transwell system, while HUVECs (1 × 10^4^ cells/well) were cultured in complete medium at the upper chamber. After 12 h, the cells attached on the upper surface of the filter membranes were gently wiped, while those migrating onto the lower surface were stained with 0.5% crystal violet for 5 min. The numbers of migrated cells were counted in three random fields using an optical microscope (Leica).

### 3.5 Tube formation assay

Tube formation in HUVECs under the co-culture with EXE-EVs or SED-EVs was assessed by a Matrigel-based capillary-like tube formation assay. Before this assay, 50 μl of Matrigel was added to each well of the 96-well plate (BD Corporation, San Jose, United States) and placed at 37°C for 1 to gel. Thereafter, HUVECs (2 × 10^4^/well) were seeded on the Matrigel and cultivated in SED-EV– or EXE-EV–contained complete medium, with 3 replicates being set under each treatment. HUVECs cultured in complete medium without EV stimulation were applied as the negative control. After 12 h of co-culture, an inverted microscope (Leica) was employed for observing tube formation. The cord-like structures were captured. Branching points and tube length were counted in three random fields using ImageJ software.

### 3.6 Elisa assays to determine vascular endothelial growth factor expression

The enzyme-linked immunosorbent assay (ELISA) method was employed to analyze the influence of circulating EVs on VEGF production. The concentrations of VEGF in the supernatants of HUVECs co-cultured with SED-EVs or EXE-EVs were measured using a VEGF (rat) ELISA Kit (K5365-100, Biovision, Milpitas, United States) according to the protocol of the manufacturer. The OD values of all wells at 450 nm were measured using a Hercules microplate reader. The protein concentration for each sample was determined based on the standard curve. HUVECs cultured in complete medium without EV stimulation were used as the negative control.

### 3.7 Fabrication and evaluation of decellularized dermal matrix hydrogel

#### 3.7.1 Preparation of decellularized dermal matrix hydrogel

Commercialized human-derived decellularized dermal matrix (DDM) was purchased from Beijing Jayyalife Biological Technology Co., Ltd. (J-1, Jayyalife, Beijing, China). Typically, DDM is approved for clinical application by the Chinese Food and Drug Administration. DDM is a dermal substitute obtained by epidermis removal and decellularization of human skin tissue. DDM is a milky-white, soft and elastic dermal graft which retains the morphology, structure, and composition of the extracellular matrix and can induce regenerative fibroblasts and vascular endothelial cells to grow into its framework. Meanwhile, the basement membrane between the epidermis and dermis is completely preserved in DDM, which plays an important role in the growth and differentiation of cells. As cellular components of the dermis are removed, DDM can be tolerated in the host without provoking an immune rejection. Additionally, DDM is sterile and free of viruses, bacteria, and spores. In this study, DDM freezing and lyophilization were performed using a vacuum freeze-drier (FD8-5T, SIM, FL, United States) for 24 h in order to prepare the hydrogel. DDM Hydrogel (DDMH) was acquired according to a protocol reported previously (Wolf et al., 2012; Lin et al., 2021). In brief, DDM was grinded into powder and sieved through a 40-mesh screen, followed by enzymatic digestion in 1 mg/ml pepsin solution (Sigma) contained within 0.01 M hydrochloric acid under constant stirring for 48 h at room temperature. The acidic digest solution was diluted with 10 × PBS to a concentration of 10 mg/ml and then neutralized by the addition of 0.1 M sodium hydroxide and 10 × PBS on ice to a pH of 7.0. Through the above steps, the neutralized solution obtained was the pre-gel. The pre-gel was stored at 4°C and taken out for gelation at 37°C for 30 min.

#### 3.7.2 Cell Viability

HUVECs were seeded on the DDMH gel (DDMH group) in 96-well plates at a density of 4 ×10^3^ cells/well (four replicates per group) and cultured in complete medium which were replaced every two days. After being incubated at 37°C for 3 days, a live/dead assay kit (40747ES76, Yeasen, Shanghai, China) was applied to stain the HUVECs cultivated on DDMH. A fluorescence microscope (AxioImager.M2, ZEISS) was utilized to capture images for green- and red-stained cells at the 488 and 594 nm excitation wavelengths, which represented live and dead cells, respectively. This work determined cell viability as follows: (live cell count/overall cell count) × 100%. Besides, CCK-8 assays were also performed using supernatants of HUVECs cultured on DDMH as described above. HUVECs cultivated on tissue culture polystyrene (TCP) without hydrogel served as the control.

#### 3.7.3 Immunogenicity of decellularized dermal matrix hydrogel

RAW264.7 cells (5.0 × 10^5^ cells/well) were seeded on DDMH in a 6-well plate (four replicates per group) and maintained with 2 ml complete medium. Meanwhile, an equal amount of RAW264.7 cells cultured on TCP with 2 ml complete medium or 2 ml complete medium containing 10 mg/ml lipopolysaccharide (LPS) were served as a negative or positive control. After culture for 3 days, supernatants were collected for detecting PGE-2, TNF-α, IL-1β, and IL-6 levels using ELISA kits (Multi Sciences).

### 3.8 Enzyme-linked immunosorbent assay to assess the influence of decellularized dermal matrix hydrogel on vascular endothelial growth factor production

ELISA assay was performed to assess the influence of DDMH on VEGF secretion of HUVECs. HUVECs at a density of 5.0 × 10^5^ cells/well (four replicates per group) were cultured on DDMH or TCP with 2 ml complete medium. After 3 days of culture, the supernatants were collected to detect the VEGF level using a VEGF (rat) ELISA Kit (K5365-100, Biovision).

### 3.9 Extracellular vesicles/decellularized dermal matrix hydrogel compound construction

SED-EVs or EXE-EVs were thawed at 4°C. 10 μL EV solution (10 μg/μL) was inoculated into 100 μL DDMH at 4°C (Wang et al., 2020). The mixture was fully blended under constant stirring for 2 h at 4°C in order to construct the EV/DDMH compound. The material was stored at 4°C while avoiding light for later use.

### 3.10 Sustained extracellular vesicle release properties of decellularized dermal matrix hydrogel

The sustained release properties of the DDMH were evaluated and compared with those of gelatin hydrogel (GH). Briefly, the EV/DDMH compound or EV/GH compound (equal amount of EVs inoculated in gelatin hydrogel) was positioned in PBS and incubated at 37°C. BCA protein assay was performed to detect the release of EVs from GH or DDMH. The supernatants were collected continuously for 14 days to detect the amount of released EVs, and a release curve was drawn.

## 4 *In vivo* assessment in a diabetic rat model

### 4.1 Rat diabetic wound model and treatment

A rat diabetic wound model was established based on the previous report (Shi et al., 2021). In brief, 6-week-old male SD rats (weighing 250–300 g) received intraperitoneal injection of a single dose of streptozotocin (75 mg/kg). Later, rats that had hyperglycemia (blood glucose > 15 mM) and showed weight loss, polydipsia, and polyuria were defined as diabetic rats, which were used for wound healing experiments. One week later, the diabetic rats were submitted to general anesthesia with 3% pentobarbital (1.2 ml/kg), followed by shaving of dorsal hair using an electric clipper. After adequate sterilization with 75% ethyl alcohol, a circular full-thickness wound with a diameter of 2 cm was made on the back. Then, the wounds were patched with three different materials, respectively, DDMH (DDMH group), DDMH containing SED-EVs (SED-EVs/DDMH group), and DDMH containing EXE-EVs (EXE-EVs/DDMH group). Thereafter, the wounds were covered with sterile wound dressing. After the operation, rats were separated to single breeding in order to prevent them from biting each other. The DDMH or EVs-DDMH patches were applied to wounds once until skin samples were harvested.

### 4.2 Extracellular vesicles tracking *in vivo*

For *in vivo* DiR-labeled EV tracking, the intensity and distribution of DiR signals were detected using a non-invasive tracking system (IVIS Spectrum, PerkinElmer, United States) at the wound sites on days 3 and 7 postoperatively.

### 4.3 Evaluation of wound closure and blood vessel regeneration of gross appearance

On days 0, 7, 14, and 21 postoperatively, photographs of wounds were taken, and these images were imported into ImageJ software to measure and calculate wound size *via* 2 independent researchers (Haifeng Liu and Bing Wu). Wound healing rates were later examined based on the formula below: (D_0_-D_t_)/D_0_ × 100%, with “D_0_” indicating the wound size at day 0 and D_t_ representing the wound size at the measured time point. To examine angiogenesis of the wound tissue, the underside of the skin at day 10 post-wounding was monitored and photographed.

### 4.4 Histological evaluation

The rats were terminated 21 days after surgery to harvest samples that contained the wound bed as well as surrounding healthy skin. After flattening, 4% neutral paraformaldehyde (PFA) was added to fix the samples for 24 h, followed by ethanol dehydration, xylene hydration, paraffin embedding, and slicing into 5-μm sections. Then, sections were stained with hematoxylin and eosin (H&E) to detect the neuroepithelium length and with Masson’s trichrome (MT) to observe the collagen synthesis. Sections were photographed using an optical microscope (Leica DMI6000B) and images were analyzed using ImageJ software. The re-epithelialization rate (E%) in H&E-stained sections was analyzed using the following formula: E% = W_t_/W_0_ ×100, with “W_0_” indicating original wound length and “W_t_” representing neo-epithelial length on wound surface (Shi et al., 2021). The mean staining intensity of Masson in MT-stained sections was calculated in 5 random visual fields per section to determine collagen maturity level within wound beds. All measurements were performed thrice by two independent researchers (Bing Wu and Haifeng Liu).

### 4.5 Microfil perfusion and X-ray microscope evaluation of blood vessel regeneration

Microfil perfusion and X-ray microscope examination were conducted to assess the neovascularization. After anesthesia with phenobarbital (3%, 1.2 ml/kg) on the 10th day after surgery, the heart was exposed by opening rat rib cages. First, an indwelling needle was placed in the left ventricle after the pulmonary artery and pulmonary vein were clamped. Subsequently, the left ventricle was injected with 100 ml heparinized saline to empty the blood vessel *via* the indwelling needle, followed by immediate perfusion with 15 ml microfil (Microfil MV-122, Flow Tech, Carver, MA, United States) in the beating left ventricle at a rate of 3 ml/min (Yu et al., 2020). To completely induce polymerization of the microfil agent, the perfused specimens were placed at 4°C for 24 h. Then, circle skin tissues with a radius of 15 mm from the wound center around the wound sites were harvested for imageological examination. An X-ray microscope (Xradia 410 Versa, Zeiss, Oberkochen, Germany) was used to scan the skin samples at a resolution of 4.5 μm to observe neo-vascularization, and Dragonfly software (Dragonfly Software, Bluffton, United States) was used for three-dimensional reconstruction of neo-vessels. Meanwhile, these images were uploaded to ImageJ software for measurement and calculation of the total vessel cover density (VCD). The circle area of each sample with a radius of 15 mm from the wound center was designated as the region of interest, where vessel coverage was carefully distinguished. VCD was calculated as follows: (A_v_)/A_0_ × 100%, where “A_0_” is deemed the total area of measurement region and “A_v_” denotes the area covered by blood vessels.

### 4.6 CD31 immunofluorescence and quantitation of microvessel density

CD31 immunofluorescence (IF) staining was performed to assess the extent of newly formed microvessels in wound-healing. In brief, 4% PFA was added to fix skin samples on day 10 after wounding, followed by dehydration with 30% sucrose solution, embedding within the optimum cutting-temperature compound (O.C.T, Sakura finetec United States inc, Torrance, United States), and slicing into 5-μm sections. OCT was rinsed, followed by 30 min of blocking using 3% BSA under ambient temperature. Then, the frozen sections were incubated with the mouse anti-CD31 antibody (1:100; ab24590, Abcam, Cambridge, Britain) overnight at 4°C and then with the goat anti-mouse IgG H&L secondary antibody (1:2000; ab150116, Abcam) at room temperature for 1 h in the dark. 0.5 μg/ml DAPI (Invitrogen) was adopted for nuclei staining. The signals were captured using a fluorescence microscope (AxioImager.M2, Zeiss), and the images were analyzed using ImageJ software. Then, 3 fields of view (FOVs, 10 × 10) were randomly selected from each section at the wound site, and blood vessel quantity was determined for every group in 3 FOVs under high magnification (10 × 20).

### 4.7 qRT-PCR analysis of angiogenesis-related genes

Quantitative reverse transcription-polymerase chain reaction (qRT-PCR) was utilized to detect the expression of angiogenesis-related genes in the wound including *VEGF* and angiopoietin-1 (*ANG1*) at day 10 post-wounding. Briefly, total RNA was extracted from the full-thickness traumatic tissue of the different groups using Trizol Reagent (Invitrogen, Carlsbad, United States). Subsequently, total RNA was converted to cDNA by reverse transcription using the Revert Aid first-strand cDNA synthesis kit (Fermentas, Burlington, Canada). Then, the ABI PRISM^®^ 7900HT System (Takara Biotechnology, Japan) was employed for real-time PCR using SYBR Premix Ex Taq™ II (Takara). The relative mRNA expression was calculated using the relative standard curve method (2^−△△CT^). Each reaction was conducted in triplicate, with GAPDH being the endogenous reference. Sequences of primers are shown as follows: *VEGF*: forward, 5′-GGG​CAG​AAT​CAT​CAC​GAA GT-3, and reverse, 5′-AAA​TGC​TTT​CTC​CGC​TCT​GA-3′; *ANG1*: forward, GAG​CAT​AAA​ATC​CTA​GAA​ATG​G, and reverse, TGC​AGA​ACA​CTG​TTG​TTG​CTG​G; *GAPDH* forward, 5′-GGA​GCG​AGA​TCC​CTC​CAA​AAT-3′, and reverse, 5′-GGC TGT​TGT​CAT​ACT​TCT​CAT​GG-3′.

### 4.8 Statistical analysis

Results were represented by means ± standard deviation. Student’s *t*-test was used for the comparison between two groups and one-way ANOVA with a *post hoc* test for the multiple group comparison. GraphPad Prism software (Version 8, San Diego, United States) was adopted for statistical analysis. *p* < 0.05 was considered statistically significant.

## 5 Result

### 5.1 Extracellular vesicle characterization and internalization

**FIGURE 1:**
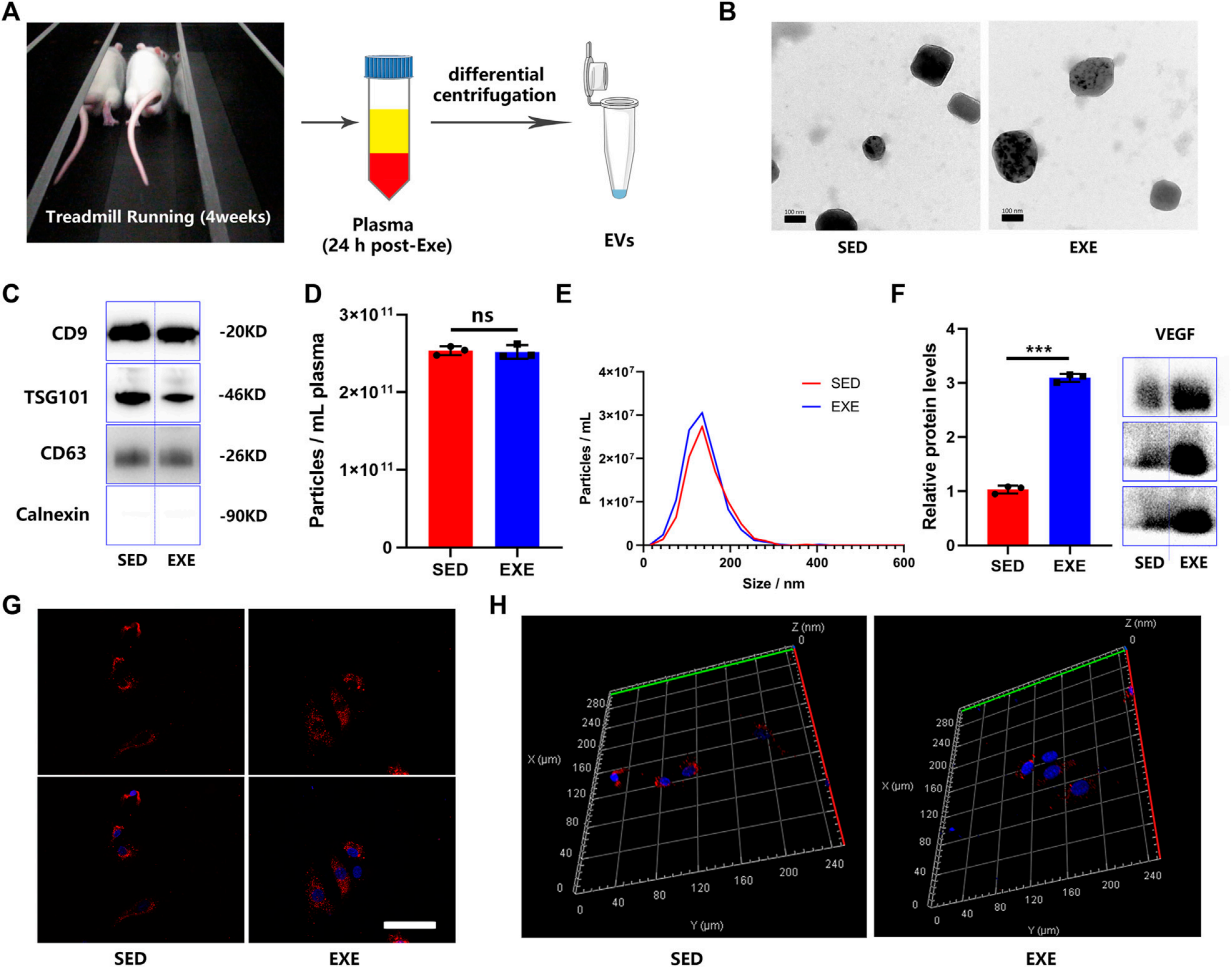
Characterization of plasma EVs from exercised or sedentary rats. **(A)** EVs were isolated 24 h after the last training session from the plasma of rats subjected to 4 weeks of running exercise or from sedentary littermates. **(B)** Morphology of plasma circulating EVs from sedentary littermates (SED-EVs) and exercised rats (EXE-EVs) under transmission electron microscopy. Scale bar: 100 nm. **(C)** Detection of the EV surface markers (CD9, TSG101m, CD63, and Calnexin) in SED-EVs and EXE-EVs. **(D,E)** Average particle concentration and size distribution of SED-EVs and EXE-EVs calculated by nanoparticle tracking analysis (*n* = 3). **(F)** Relative VEGF levels in SED-EVs and EXE-EVs. **(G,H)** HUVECs were co-cultured with PKH26 labeled SED-EVs or EXE-EVs. EVs were observed be internalized by HUVECs as indicated by immunofluorescence images **(G)** and 3D reconstruction of the z-stack images **(H)**. Nuclei were stained with DAPI (blue). Scale bar: 50 μm ***p < 0.001.

EVs were isolated from the plasma of exercised rats (EXE-EVs) and sedentary rats (SED-EVs) 24 h after the last training session (Figure 1A). The obtained EVs showed a typical cup-like appearance with double membrane structures extending from 50 to 200 nm under TEM (Figure 1B). WB analysis verified the presence of extracellular vesicle marker proteins (CD9, TSG101, and CD63) (Figure 1C). In addition, NTA presented no obvious differences in plasma concentration (2.54 × 10^11^ ml^−1^ vs. 2.52 × 10^11^ ml^−1^) or size distribution between SED-EVs and EXE-EVs (Figures 1D,E). Moreover, VEGF was found to be present in both SED-EVs and EXE-EVs, while VEGF levels in EXE-EVs were significantly higher than that in SED-EVs (*p <* 0.001, Figure 1F). After 6 h of incubation, PKH26-labeled EVs were internalized by HUVECs as suggested by immunofluorescence images (Figure 1G) and a 3D reconstruction of the z-stack images (Figure 1H).

### 5.2 Exercise-induced circulating extracellular vesicles promote endothelial cell proliferation and migration

**FIGURE 2:**
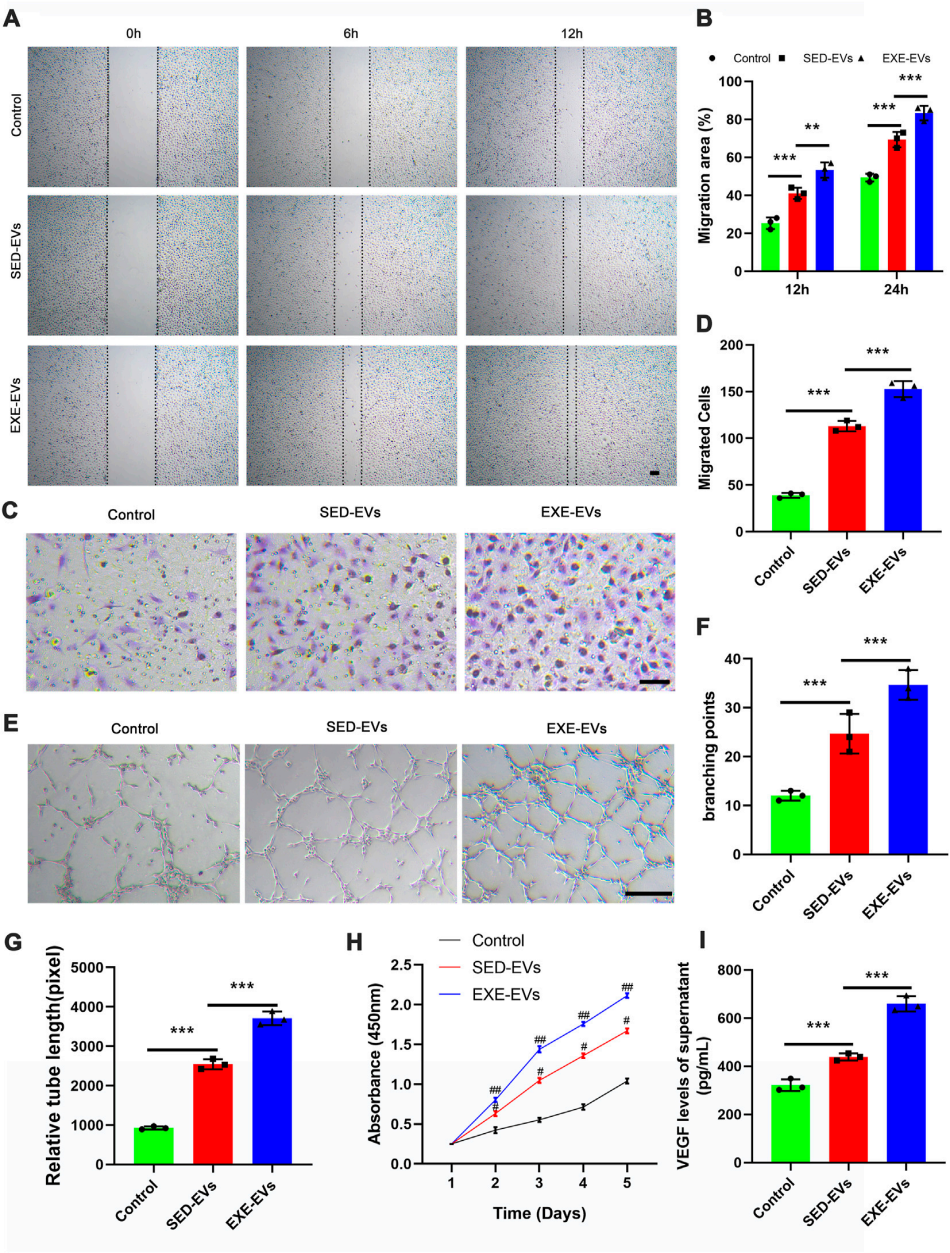
EXE-EVs promote proliferation, migration, and angiogenic activities of endothelial cells. **(A)** EXE-EVs promoted HUVECs migration as analyzed by scratch wound assay. Scale bar: 50 μm. **(B)** Quantitative analysis of the migration area in **(A)**. *n* = 3 per group. **(C)** The migratory ability of HUVECs receiving EXE-EVs treatments was further confirmed by the transwell assay. Scale bar: 100 μm. **(D)** Quantitative analysis of the migrated cells in **(C)**. *n* = 3 per group. **(E)** More formation of capillary-like structures was observed under the stimulation of EXE-EVs. Bar = 100 mm. **(F,G)** Quantitative analysis of the total branching points **(F)** and total tube length **(G)** in **(E)**. *n* = 3 per group. **(H)** The proliferation of HUVECs receiving different treatments was assessed by CCK-8 analysis. *n* = 3 per group. **(I)** The VEGF concentration of supernatants from SED-EV– or EXE-EV– treated HUVECs *n* = 3 per group. #*p* < 0.01 compared with the control group. ##*p* < 0.01 compared with the SED-EVs group. **p < 0.01, ***p < 0.001.

Both scratch wound assay and transwell assay were employed to decide the impact of circulating EVs on the migration of HUVECs. The result showed that EXE-EV treatment obviously stimulated the motility of HUVECs, as determined by the migration area and migrated cells (Figures 2A,C). Quantitatively, the EXE-EVs group showed significantly larger wound closure area and more migrated cells than the SED-EVs group (Figures 2B,D, *p* < 0.001 for all). These data indicated that EXE-EVs exhibit better pro-migratory ability of HUVECs than SED-EVs did. Meanwhile, CCK-8 assays revealed that EXE-EVs treatment significantly enhances HUVECs proliferation compared to the other two groups (*p* < 0.001 for all, Figure 2H).

### 5.3 Exercise-induced circulating extracellular vesicles improved angiogenic activities of endothelial cells

The ability of EXE-EVs to stimulate angiogenesis *in vitro* was assessed by using the endothelial tube formation assay. As depicted in Figures 2E,F,G, the total branching points and relative tube length at the indicated time were remarkably increased in the EXE-EVs–treated HUVECs in comparison with those in SED-EVs–treated HUVECs (*p* < 0.001). In addition, EXE-EV stimulation could significantly increase the VEGF level of the HUVECs supernatant when compared with the SED-EVs group and the control group (*p* < 0.001 for all, Figure 2I)

### 5.4 Decellularized dermal matrix hydrogel has thermosensitive properties with low immunogenicity and no cytotoxicity

**FIGURE 3:**
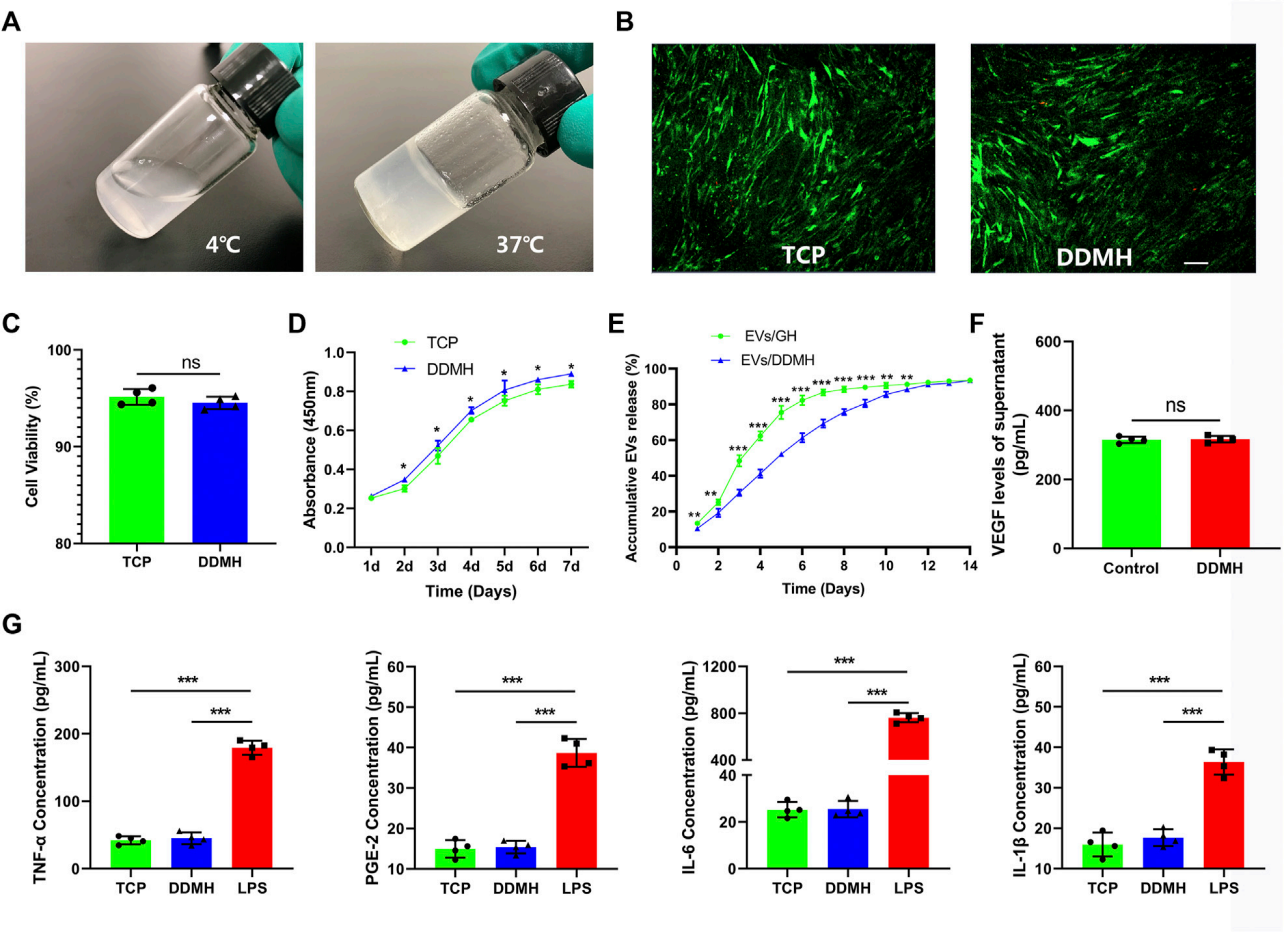
Evaluation and characterization of DDMH. **(A)** DDMH is in a liquid state at 4°C and in a gel state at 37°C. **(B)** HUVECs were inoculated and cultured with DDMH or TCP, and cell viability was detected with live/dead staining on the 3rd day. Scale bar: 100 μm **(C)** Quantitative analysis of cell viability. *n* = 4 per group. **(D)** CCK-8 assay was used to detect the proliferative capability of HUVECs seeded on DDMH and TCPs. *n* = 4 per group. **(E)** EVs released from DDMH or GH with time. **(F)** VEGF released from DDMH was detected by ELISA assay. **(G)** Immunogenicity of DDMH was detected by ELISA analysis of TNF-α, PGE-2, IL-6, and IL-1β levels. *n* = 4 per group. *p < 0.05, **p < 0.01, ***p < 0.001.

DDMH which has thermosensitive property was successfully acquired from commercialized DDM. DDMH pre-gel is liquid at low temperatures (4°C) and transforms into a gel state at 37°C (Figure 3A), which makes it an injectable scaffold material for skin defect repair. Cell viability of HUVECs seeded on the gel was detected on the third day of co-culture to determine cytotoxicity of DDMH. The results showed that culture with DDMH did not increase dead cell numbers (*p* = 0.28, Figures 3B,C), indicating that DDMH has no cytotoxicity. Meanwhile, HUVECs seeded on DDMH showed enhanced proliferation when compared with those cultured on TCPs (*p* < 0.05 for all, Figure 3D). Additionally, the supernatants of the TCP and DDMH groups revealed similar levels of pro-inflammatory cytokines (TNF-a, PGE-2, IL-6, and IL-1β), which were significantly lower than those in the LPS group (*p* < 0.001 for all Figure 3G). The obtained findings indicated that DDMH is a biomaterial scaffold with low immunogenicity.

### 5.5 Decellularized dermal matrix hydrogel has no influence on vascular endothelial growth factor expression of endothelial cells

Supernatants of HUVECs cultured on the TCP and DDMH showed similar levels of VEGF (*p* = 0.75, Figure 3F). The data suggested that the DDMH does not increase the production of VEGF in HUVECs.

### 5.6 Sustained release of extracellular vesicles by extracellular vesicle/decellularized dermal matrix hydrogel compound

As shown in Figure 3E, the release of EVs from DDMH is more gentle and durable than that from the GH group. GH released over 50% EVs in the first 3 days and nearly 80% EVs in the first 5 days. By contrast, DDMH released 30.53% EVs in the first 3 days, no more than 60% in the first 5 days, and about 70% by the 7th day. The release steadily proceeded until the 12th day in the EV/DDMH compound. These data confirmed the sustained EV release properties of DDMH.

### 5.7 Extracellular vesicles tracking *in vivo*

**FIGURE 4:**
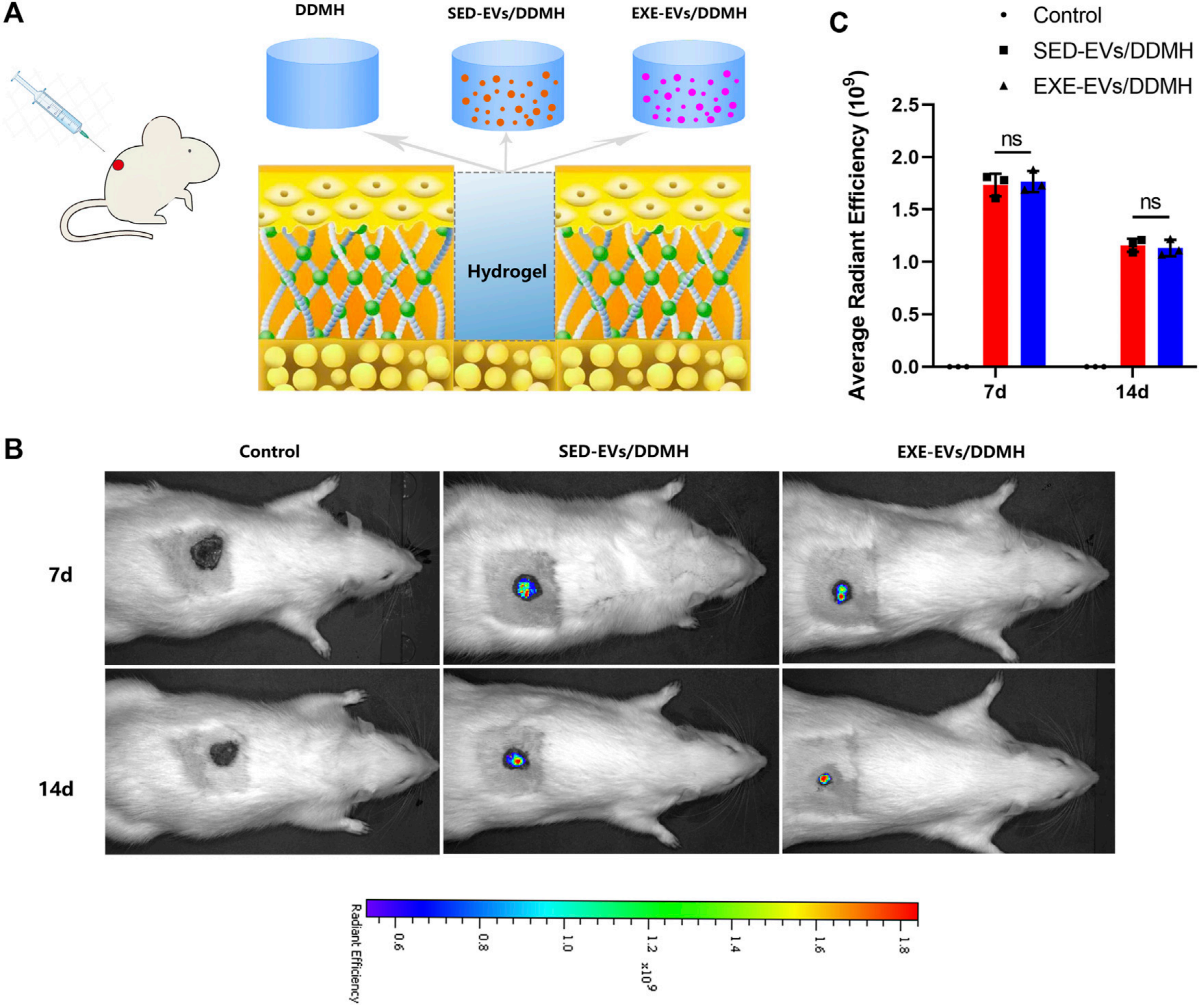
Treatment of rat diabetic wound and EV tracking *in vivo*. **(A)** Schematic illustrating the application of DDMH, SED-EVs/DDMH, or EXE-EVs/DDMH in diabetic wound healing. **(B)** A non-invasive *in vivo* fluorescence tracking analysis demonstrated that DiR-labeled EVs were delivered to the targeted area. **(C)** Semi-quantification analysis showed no significant difference of mean fluorescence intensity between the SED-EVs/DDMH group and EXE-EVs/DDMH group. *n* = 3 per group.

As illustrated in Figure 4A, rat diabetic wounds were patched with DDMH, SED-EVs/DDMH, and EXE-EVs/DDMH, respectively. After injection of DiR-labeled EXE-EVs or SED-EVs at wounds for 3 and 7 days, IVIS images demonstrated that the DiR signal persistently existed in the wounds and decreased with time and that the average immunofluorescence intensity in the EXE-EVs/DDMH group and the SED-EVs/DDMH group showed no significant difference (*p* = 0.90, Figures 4B,C). The data indicated that the labeled EVs were delivered to the targeted area.

### 5.8 Exercise-induced circulating extracellular vesicles/decellularized dermal matrix hydrogel accelerates cutaneous wound healing in diabetic rats

**FIGURE 5:**
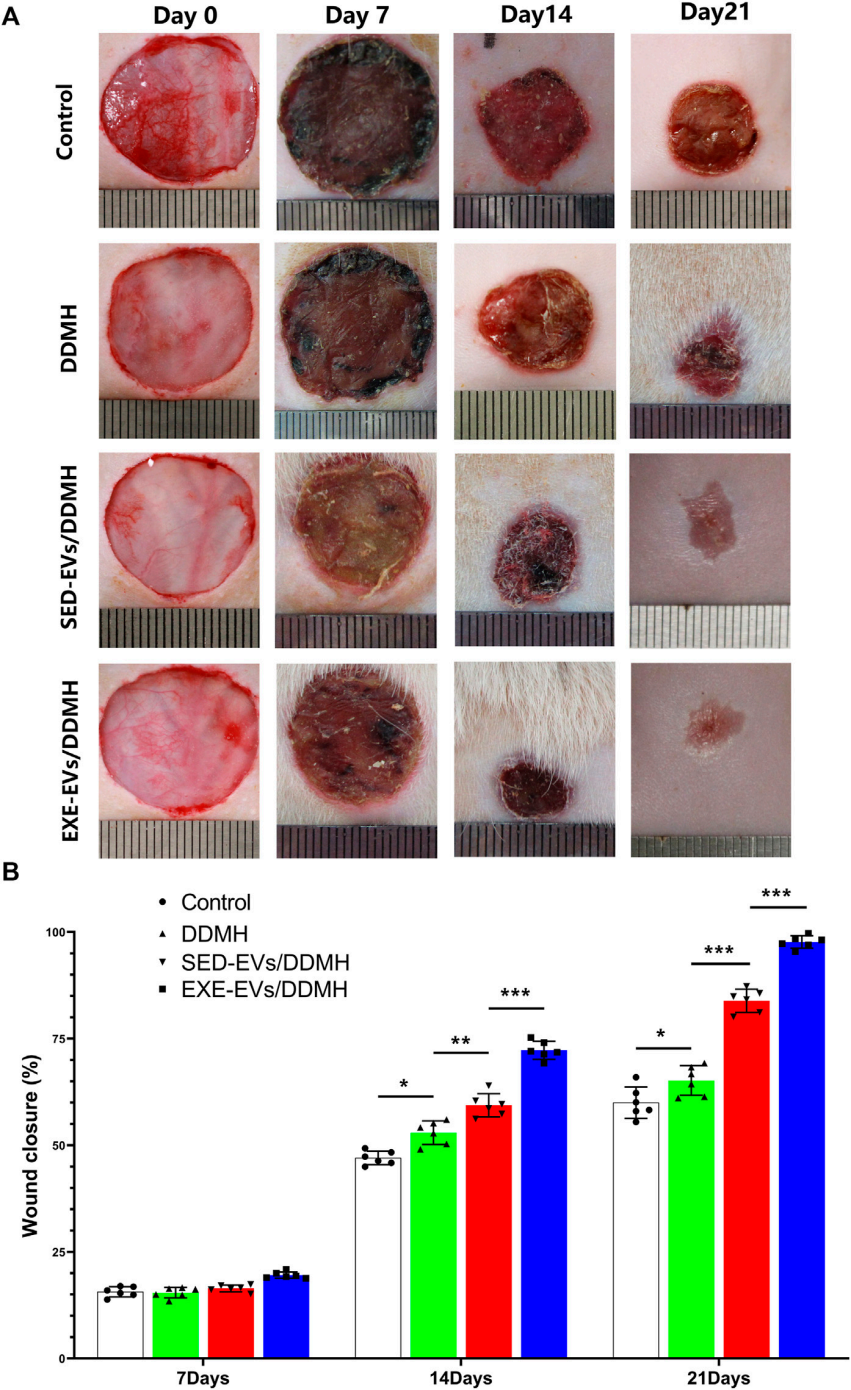
EXE-EVs/DDMH accelerates cutaneous wound healing in diabetic rat. **(A)** Overview of the size change of the wounds made in the dorsal skin of diabetic mice among the four groups at postoperative days 0, 7, 14, and 21. Scale bar: 1 cm **(B)** The wound closure rate of the four groups at the indicated times. *n* = 6 per group. *p < 0.05, **p < 0.01,***p < 0.001.

As shown in Figure 5A, no sign of inflammation or infection was found in the wounds at varying time points. Wound area reduced gradually with new epidermis growing from the edge of the wound. At day 7 post-wounding, the wound closure rates in the control, DDMH, and SED-EVs/DDMH groups were similar without obvious difference, but all were significantly lower than that of the EXE-EVs/DDMH group (Figure 5B). At day 14 and day 21, DDMH and SED-EVs/DDMH intervention showed much faster wound closure in diabetic rats in comparison with the control group (*p* < 0.001 for all), while EXE-EVs/DDMH treatment exhibited significant smaller wound areas when compared with the SED-EVs/DDMH and DDMH treatment (Figure 5B).

**FIGURE 6:**
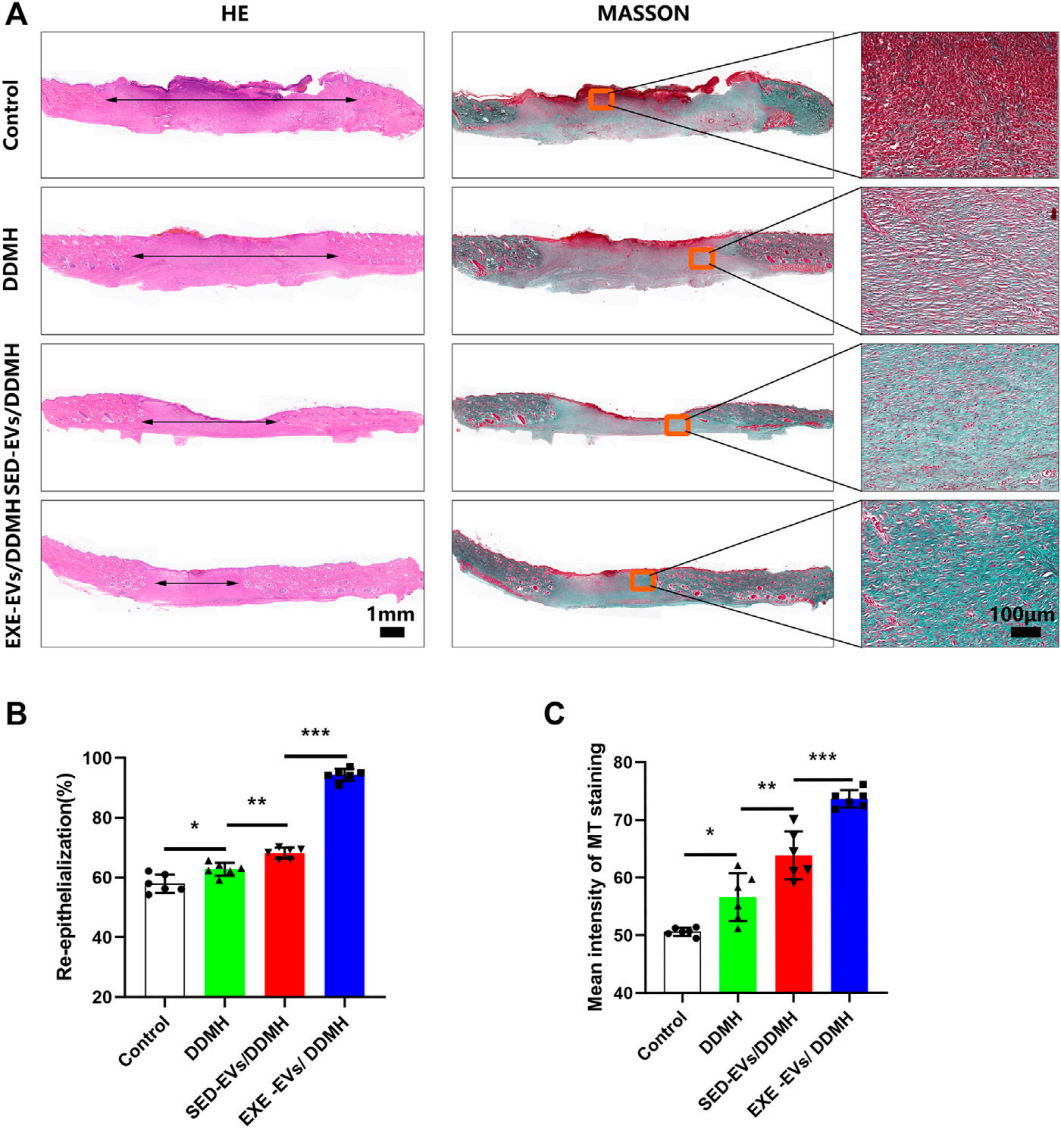
EXE-EVs/DDMH promote re-epithelialization and collagen synthesis **(A)** H&E and Masson’s trichrome (MT) staining of wound sections in the control, and DDMH, SED-EVs/DDMH, or EXE-EVs/DDMH groups at 21 days post-wounding. The double-headed black arrows indicate the edges of the scars. Bar = 100 μm. **(B,C)** Quantification of the re-epithelialization extent **(B)** and the mean intensity of MT staining **(C)** in histological sections of the four groups. *n* = 5 per group. *p < 0.05, **p < 0.01, ***p < 0.001.

Histologically, re-epithelialization degree was analyzed by H&E staining. As depicted in Figure 6A, wounds treated with DDMH or EVs/DDMH exhibited extended neo-epidermis and dermis with regenerated hair follicles and fat cells compared to that of the blank group on day 21 after wound formation. Quantitatively, wounds treated with EXE-EVs/DDMH showed a higher rate of re-epithelialization than other therapies (*p* < 0.001 for all, Figure 6B). Meanwhile, collagen deposition and maturation were evaluated by MT staining. At day 21, increased collagen deposition and thick wavy collagen fibers were observed in the EXE-EVs/DDMH and SED-EVs/DDMH groups (Figure 6A). According to quantitative analysis, the EXE-EVs/DDMH group showed significantly larger intensity of MT staining than SED-EVs/DDMH and the other two groups. (*p* < 0.001 for all, Figure 6C). These data indicated that EXE-EVs/DDMH effectively enhances diabetic wound repair.

### 5.9 Exercise-induced circulating extracellular vesicles/decellularized dermal matrix hydrogel stimulates angiogenesis in diabetic wounds

**FIGURE 7:**
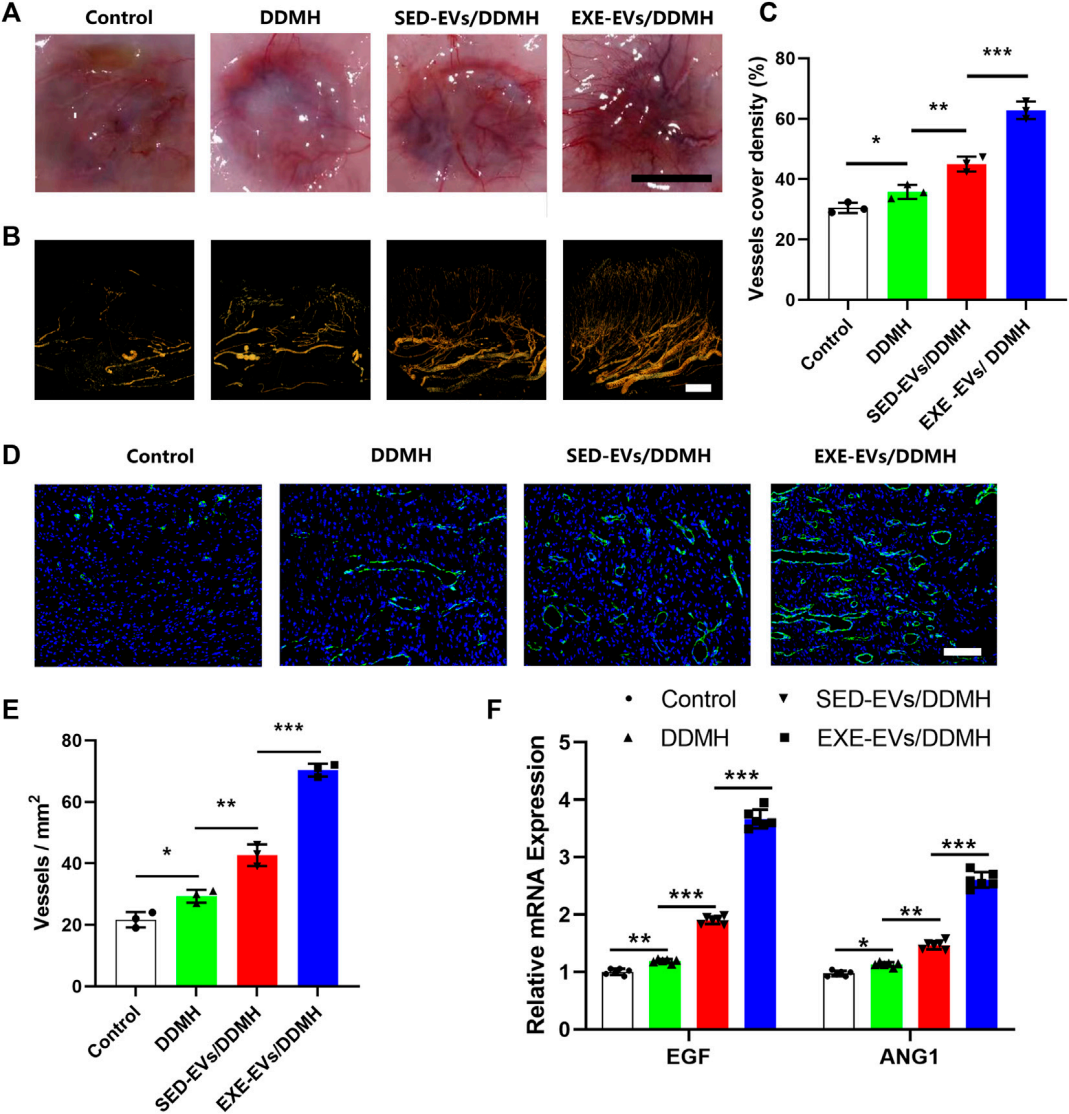
EXE-EVs/DDMH promotes angiogenesis in the wound sites of diabetic rats. **(A)** Gross appearance of the newly formed vessels at the underside of skin 10 days after surgery. Scale bar: 1 cm **(B)** Three-dimension vessel images of the wound site in the four groups by microfil perfusion. Bar = 2 mm. **(C)** Quantification of the vessels cover density in **(B)**. *n* = 3 per group. **(D)** Immunofluorescent staining for CD31 in wounds covered with DDMH, SED-EVs/DDMH, or EXE-EVs/DDMH at day 10 post-wounding. Bar = 100 μm. **(E)** Quantitative analysis of the number of total blood vessels in wounds at day 10 post-wounding. *n* = 3 per group. **(F)** mRNA expression of VEGF and ANG1 in the wound under SED-EVs/DDMH or EXE-EVs/DDMH treatment. *n* = 6 per group. *p < 0.05, **p < 0.01, ***p < 0.001.

Considering that EXE-EVs exhibited superior endothelial inducibility *in vitro*, we evaluated neovascularization at the wound site to explore the efficacy of EXE-EVs/DDMH on the stimulation of angiogenesis. As shown in Figure 7A, much more newly formed blood vessels were found in the wounds exposed to treatment with EXE-EVs/DDMH at day 10 post-wounding in comparison with other groups. Additionally, three-dimension vessel images of the wound site by microfil perfusion suggested that wounds treated with EXE-EVs/DDMH significantly enhanced neovascularization, which was determined by larger vessel cover density (*p* < 0.001 for all, Figures 7B,C). Furthermore, immunofluorescence staining for CD31 was also carried out in order to identify the extent of blood vessel formation in the wound sites. As shown in Figure 7D, the blood vessels were rarely observed in the control group and the DDMH group, whereas SED-EVs/DDMH– or EXE-EVs/DDMH–covered wounds significantly increased the number of CD31 positively stained cells. Meanwhile, more blood vessels were identified in the EXE-EVs/DDMH–covered wounds than in the SED-EVs/DDMH–covered wounds (*p* < 0.001, Figure 7E). Besides, mRNA expression of angiogenesis-related genes including *VEGF* and *ANG1* in the EXE-EVs/DDMH group was remarkably elevated compared to the SED-EVs/DDMH group and the control group (*p* < 0.001 for all, Figure 7F). All these data indicated that EXE-EV transplantation augments the angiogenic responses of the wound sites in diabetic rats.

## 6 Disscussion

The present study offered the first demonstration that EXE-EVs sustained release by DDMH-induced prominent angiogenic effects in diabetic wound healing, as defined by more rapid wound closure, higher rates of re-epithelialization, and more collagen deposition. In addition, we also revealed *in vitro* that EXE-EVs could be internalized into endothelial cells and enhance their functional properties of proliferation, migration, capillary-like tube formation, and angiogenesis-related gene expression. Our results suggest that EXE-EVs combined with DDMH may serve as a new cell-free tissue engineering therapy agent for enhancing diabetic wound repair and regeneration.

Numerous evidence has demonstrated that angiogenesis plays a vital role in all wound healing stages (Carmeliet and Jain, 2011; Ahluwalia and Tarnawski, 2012; Lancerotto and Orgill, 2014; DiPietro, 2016; Azari et al., 2022) Hypoxia environment following injury can activate hypoxia-inducible factor-1 (HIF-1) and then trigger the expression of downstream VEGF, ANG-1, bFGF, and other pro-angiogenic factors which guide vascular growth from the wound periphery into the wound bed (Broughton et al., 2006; Liu et al., 2008). In contrast to normal wound healing, the diabetic disease can significantly decrease angiogenesis in healing wounds, which disrupts tissue regeneration and usually leads to chronic non-healing wounds (Dinh and Veves, 2005; Falanga, 2005; Brem and Tomic-Canic, 2007). Hence, we focus on the neovascularization of wounds and choose to detect the level of the key angiogenesis factors VEGF and ANG-1 to determine the pro-angiogenic efficiency of exercise-derived EVs *in vivo*. The present study has shown that both pro-angiogenic mediators and neovascularization significantly decreased in diabetic wounds treated with saline. Several factors might attribute to this pathological change. First, macrophages in diabetic wounds usually fail to switch from the pro-inflammatory to the pro-reparative phenotype, which significantly decreases macrophages release of the cytokines such as VEGF (Galiano et al., 2004; Mirza and Koh, 2011). Besides, the diabetic state leads to an inherently decreased population of endothelial progenitor cells (EPCs) from the bone marrow, which can also reduce the baseline vascularity in diabetic tissues (Drela et al., 2012). Given the many changes in pro-angiogenic and vascular maturation factors in diabetes, it is crucial to enhance angiogenesis of diabetic wounds.

Inactivity is a recognized risk factor involved in the development of diabetes mellitus. Previous studies performed in both humans and animal models of diabetes mellitus demonstrated that exercise confers systemic benefits on improved glycemic control (Madsen et al., 2015) as well as insulin sensitivity (Mikines et al., 1988; Martin et al., 1995) and minishing complications (Colberg et al., 2016). Meanwhile, some studies also support beneficial effects of exercise on endothelial and smooth muscle function (Maiorana et al., 2001; Cohen et al., 2008; Colberg et al., 2010a; Martin et al., 2012; Mitranun et al., 2014). Additionally, it has been verified that regular exercise training increases microvascular and capillary density, enhances capillary perfusion, and improves microvascular vasodilatory function in the setting of diabetes mellitus (Colberg et al., 2010a; Naylor et al., 2016; Lanting et al., 2017). The joint-position stand from the American College of Sports Medicine (ACSM) and the American Diabetes Association (ADA) recommend regular exercise as a safe and effective therapy to manage diabetes. To be specific, guidelines suggest that a moderate aerobic exercise (corresponding to 40%–60% maximal aerobic capacity) should be conducted in bouts of 10 min or longer, at least 3 days per week and at least 150 min/week (Colberg et al., 2010b). Despite the benefits of regular physical activity to the overall health and alleviation of complications in diabetic patients, growing evidence has demonstrated that weight-bearing exercise or physical activity including walking, running, jumping, and cycling may also cause diabetic foot ulceration (DFU) or delaying ulcer healing (Lazzarini et al., 2019; Hulshof et al., 2020). Neuropathy in diabetic patients usually results in insufficient plantar protective sensation, gait abnormalities, and foot deformities, which can cause excessive levels of mechanical stress as peak plantar pressures and total loading time increase whereas shock absorption decreases (Wrobel and Najafi, 2010; Armstrong et al., 2017; Schaper et al., 2017; Lazzarini et al., 2019). The elevated levels of mechanical load in insensate neuropathic plantar tissue can contribute to subdermal trauma and inflammation, ultimately leading to ulcer development and delay ulcer healing (Frykberg et al., 1998; Pham et al., 2000; Monteiro-Soares et al., 2012). As a result, offloading techniques like non-removable knee-high devices or walkers, forefoot offloading shoes, cast shoes and custom-made temporary shoes are recommended to limit or refrain from weight-bearing activity by the International Working Group on the Diabetic Foot (IWGDF; www.iwgdf.org) (Bus et al., 2016). The contradiction between the benefits such as improvement of blood supply and unfavorable effects like increasing the plantar stress and the risk of DFU make many common types of exercise and physical activity unusable for diabetic patients with DFU or at risk of DFU. In such cases, we sought to explore alternative biotherapy for diabetic wound defect which can not only mimic beneficial effects of exercise including improving endothelial function and blood perfusion but also avoid plantar load increase.

Emerging evidence suggests that exercise training releases circulating EVs containing a unique profile of exerkines which transfer to target organs where they exert beneficial systemic effects (Safdar et al., 2016; Bertoldi et al., 2018; Fiuza-Luces et al., 2018). It has been reported that exercise-induced circulating EVs can protect against cardiac ischemia-reperfusion injury (Bei et al., 2017; Hou et al., 2019). Although EVs have been postulated to mediate the benefits of exercise in type 2 diabetes mellitus (Safdar et al., 2016; Li et al., 2019), whether exercise-induced circulating EVs exert beneficial effects on diabetic wound healing remains unknown. For the first time, the present study proved that exercise-induced circulating EVs from healthy individuals significantly enhanced endothelial cell function and promoted diabetic wound angiogenesis and healing. The current work adds evidence for circulating EVs to function as an important mediator to spread exercise-induced beneficial effects. Of note, exercise-induced circulating EVs from diabetic rats were not chosen for therapy in the present study. This is due to concerns that weight-bearing exercise like treadmill running would increase the risk of DFU development and delay ulcer healing. It can be reasonably assumed that treadmill exercise is not clinically feasible for patients with DFU. In the future, the effect of circulating EVs from human and animal models with specific exercise training that can avoid weight-bearing and wound ulcer infection could be further observed.

Interestingly, although the current research studies support the idea that both resistance and aerobic exercise can exert these beneficial effects, the latter mediates greater improvements in cutaneous microvascular endothelial function (Cohen et al., 2008; Colberg et al., 2010a; Olver and Laughlin, 2016). In the present study, we adopted the aerobic exercise program on account of the original intention of maximizing the pro-angiogenic benefits for diabetic wounds healing. Herein, based on the aforementioned aerobic exercise guidelines from ACSM and previous animal exercise models which have been reported to yield protective effects in normal aging (Bertoldi et al., 2018) and myocardial ischemia injury (Hou et al., 2019), we used a moderate intensity (60% VO_2 peak_) continuous aerobic protocol to explore the corresponding influence on angiogenetic efficiency of circulating EVs. In the present study, 4 weeks of moderate aerobic treadmill training did not alter the number of EVs in plasma, which is in line with previous reports (Whitham et al., 2018; Hou et al., 2019). In contrast, some data suggest that an acute bout of exercise can promote rapid release of EVs or exosomes into circulation (Frühbeis et al., 2015; Bei et al., 2017). The difference is probably ascribed to the exercise parameters and plasma sampling time. Frühbeis *et al* (Frühbeis et al., 2015) and Bei et al. (2017) detected an increased number of plasma EVs following an acute running protocol, whereas Hou et al. (2019) found that 1 year of rowing training or 4 weeks of swimming did not trigger statistically significant changes in EV amount. On the other hand, since the number and content profile of EVs isolated immediately after exercise may be influenced by stress-related factors (Hou et al., 2019), we investigated the long-term effects of aerobic exercise on plasma EVs instead of the acute and transient impacts of exercise. Importantly, although the level of EVs was unchanged 24 h after the last training, EVs isolated from the plasma of exercised rats exerted a significant promotion of angiogenic responses including endothelial cell proliferation, migration, and capillary formation *in vitro* and neovascularization of diabetic wounds *in vivo* compared with sedentary rats. By contrast, sedentary circulating EVs slightly but significantly enhanced endothelial cell function and wound angiogenesis as compared to the PBS group. These findings are consistent with our results that pro-angiogenic factor VEGF was present in sedentary circulating EVs, and VEGF expression in circulating EVs was further enhanced by a four-week aerobic treadmill exercise. Thus, it seems that basal VEGF content in sedentary circulating EVs might not be sufficient to promote the angiogenesis of endothelial cells. This may be an important mechanism to explain the function difference between EXE-EVs and the SED-EVs.

Obviously, these remarkable health benefits provided by exercise are complex and multi-factorial (Pedersen et al., 2001; Warburton et al., 2006). One of the most commonly accepted theories that explains exercise-mediated multi-systemic effects is the exercise-promoted cytokines or collectively termed “exerkines” released by skeletal muscles. In addition to the classical secretory pathway, exerkines have also been proved to be secreted into circulation in the form of EVs (Choi et al., 2015). In fact, the secretion of EVs has been identified as an evolutionarily conserved process increasingly appreciated as an essential mechanism of tissue/organ cross-talks communication (Egan et al., 2016). As we know, skeletal muscle accounts for 40% of the human body weight; exercise can dramatically change its metabolic profile, resulting in expression level change of over 300 muscle-derived factors (Hartwig et al., 2014). Among these exerkines, VEGF, which is recognized as the main promoter of endothelial cell migration and tubular network formation in wound healing, has been investigated for therapeutic potential (Gustafsson et al., 2001; Alexander et al., 2012). VEGF has been verified to be absolutely essential for both vascular development (Carmeliet et al., 1996; Gerber et al., 1999) and vasculature maintaining (Carmeliet, 2000). Unfortunately, VEGF has been shown to be deficient in experimental and clinical diabetic wounds (Frank et al., 1995). Hence, we detected VEGF expression in EXE-EVs and SED-EVs and found that 4 weeks of aerobic exercise significantly increases VEGF production in circulating EVs. As the functions of EVs are based on the origin and status of cells or tissues which affect EV cargoes, the pro-vascularization effect of exercise-induced circulating EVs might attribute to VEGF and other potential beneficial exerkines encapsulated in EVs and released into the circulation.

Another possible mechanism accounting for the prominent pro-angiogenetic potential of exercise-induced circulating EVs may lie in the paracrine function change of circulating angiogenic cells (CACs). Endothelial progenitor cell-derived EVs (EPC-EVs) from the plasma of moderate exercise mice have been demonstrated to enhance angiogenesis and mitigate apoptosis of endothelial cells through the SPRED1/VEGF pathway (Ma et al., 2018). The effect of exercise on EPC-EVs was taken into account because the circulating EPC pool is considered as a mirror of cardiovascular health and most of these cells act through paracrine pathways (Di Santo et al., 2009; Rigato et al., 2015). More recently, investigators have expanded the population of EPCs to include bone marrow–derived myeloid precursors, termed CACs (Kinnaird et al., 2004). Recent evidence suggests that conditioned media of CACs from individuals engaging in regular exercise training induced longer, more complex endothelial tubes *in vitro* than those of the inactive individuals (Landers-Ramos et al., 2015; Landers-Ramos et al., 2017). Collectively, exercise-induced circulating EVs may function as a collection of pro-angiogenic cargoes from both muscle-derived exerkine-contained EVs and CACs-EVs which could significantly promote the angiogenic function of endothelial cells.

In addition, the present data showed that collagen synthesis and re-epithelialization of EXE-EVs–treated diabetic wounds significantly increase with better collagen maturity scores and wound closure compared with those of SED-EVs. This is likely owing to angiogenesis supporting and intersecting with the other ongoing proliferative and remodeling activities (Reinke and Sorg, 2012). It is known that fibroblast migration, proliferation, collagen synthesis, and epithelial proliferation all occur during the time of angiogenesis (Carmeliet, 2000; Lancerotto and Orgill, 2014). New capillaries respond to the oxygen and nutrient needs of the proliferating fibroblasts and epithelial cells, while stimulated epithelial cells yield VEGF to spur the capillary growth in turn. These processes might support one another with angiogenesis playing a key role in accelerating wound healing.

In this study, a commercialized decellularized dermal matrix (DDM) was employed to prepare the injectable thermosensitive gel scaffold material according to the protocol put forward by Matthew T. Wolf (Wolf et al., 2012). DDM allografts are proposed to be an optimal skin substitute due to their biocompatibility and tissue-like behaviors (Won et al., 2019). DDM scaffolds are prepared in various forms including sheets, powders, and hydrogels in tissue repair. In the present study, DDM hydrogel was chosen for repairing skin defects due to its biocompatibility and tissue-like behaviors. Hydrogel offers benefits including injectability, the ability to fill an irregularly shaped space and penetrate into the wound bed, and the inherent bioactivity of the native matrix (Wolf et al., 2012). Importantly, biocompatible hydrogels provide a practical option for delivering large quantities of EVs or exosomes to the target site. Recently, hydrogels have attracted more and more attention on account of their crucial roles as carriers of EVs or exosomes in regenerative medicine. Shi et al. (2017) suggested that the integration of gingival MSC-derived exosomes in the chitosan/silk hydrogel sponge could significantly accelerate the healing of diabetic skin wounds. In line with another work by Zhang et al. (2018), the present study confirmed that EVs delivered in DDMH enhance the *in vivo* stability and retention of the EVs which induced valid angiogenesis. The results implied that DDMH could serve as an optimal EV encapsulation matrix by remarkably augmenting the stability of EV cargoes and increasing the half-life of EVs *in vivo*.

The present study has some limitations. First, although our data demonstrated that VEGF is significantly upregulated in EXE-EVs and indicated EXE-EVs as a potent pro-angiogenic agent, the content of EXE-EVs and SED-EVs has not been fully revealed in the present study. There might be other mechanisms accounting for the improved diabetic healing condition except for the pro-angiogenic effect of EXE-EVs. Detecting key cargoes of EXE-EVs including miRNAs, proteins, and other bioactive molecules and downstream target genes and signals is the focus of further study. Second, although the moderate-intensity continuous aerobic protocol used in our study has been proved to yield multi-systemic benefits in many studies, the fact that EV cargoes and biological function were determined by the state of the donor cells indicates that the effect of different exercise parameters on circulating EVs, such as modalities, frequency, and time window, need to be further elucidated. Third, since no model animals or *in vivo*–specific pharmacological inhibitors have yet been developed to block EV secretion, it is currently impossible to perform *in vivo* loss-of-function studies on circulating EVs in exercise-induced diabetic wound healing. Last but not least, whether exercise itself or exercise-induced circulating EVs from diabetic individuals improve diabetic wound healing has not been validated in the present study. In the future, the effect of circulating EVs from human and animal models with specific exercise training that can avoid weight-bearing and wound ulcer infection could be further observed.

## 7 Conclusion

Circulating EVs from long-term moderate aerobic exercise can effectively enhance diabetic wound healing by promotion of angiogenesis, collagen synthesis, and re-epithelialization. Our results offer a new perspective for the treatment of a diabetic wound as a novel cell-free therapy.

## Data Availability

The original contributions presented in the study are included in the article/Supplementary Material; further inquiries can be directed to the corresponding authors.
